# Crystal structure and Hirshfeld surface analysis of 4-{[(*E*)-4-(hept­yloxy)benzyl­idene]amino}-*N*-(naphthalen-2-yl)-1,3-thia­zol-2-amine

**DOI:** 10.1107/S2056989020007951

**Published:** 2020-06-16

**Authors:** Ropak A. Sheakh Mohamad, Hashim J. Aziz, Wali M. Hamad

**Affiliations:** a Salahaddin University, College of Science, Department of Chemistry, Erbil, Iraq; b Salahaddin University, College of Education, Department of Chemistry, Erbil, Iraq; c Koya University, Faculty of Science and Health, Department of Chemistry, Koya, Iraq

**Keywords:** crystal structure, heterocyclic compound, 2-amino­thia­zole, Schiff base, Hirshfeld surface analysis

## Abstract

In the title compound, C_27_H_28_N_2_OS, the naphthalene unit is planar to within 0.015 (2) Å and makes a dihedral angle of 14.24 (16)° with the thia­zole ring. The anisole ring is inclined to the thia­zole ring by a dihedral angle of 13.18 (23)°. The torsion angle between the heptyl chain and the anisole ring is 61.1 (4)°. These dihedral and torsion angles render the mol­ecule non-planar. In the crystal, mol­ecules are linked by C—H⋯π inter­actions, forming zigzag chains that propagate parallel to the *b* axis. The roles of the various inter­molecular inter­actions in the crystal packing were clarified by Hirshfeld surface analysis; the most important contributions are from H⋯H (51.5%) and C⋯H/H⋯C (31.8%) contacts.

## Abstract 2

Charge-assisted C—H⋯π hydrogen bonds along with π–π inter­actions stabilize the crystalline state. Inter­molecular inter­actions are qu­anti­fied by Hirshfeld surface analysis.

## Chemical context   

Schiff bases, *i.e*. compounds containing the azomethine group (–CH=N– or >C=N–), are important because of their physiological and pharmacological properties. They are typically synthesized by the condensation of primary amines and active carbonyl groups. The pharmacological activities of Schiff bases include anti-bacterial, anti-fungal, anti-cancer and anti-viral properties (Wang *et al.*, 2001 ▸; Yadav & Singh, 2001 ▸).
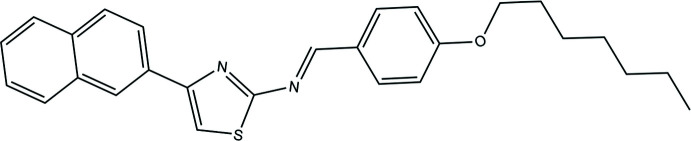


One of the most important scaffolds in drug design and heterocyclic chemistry is thia­zole, which is widely found in various pharmacologically active substances and in some naturally occurring compounds (Ayati *et al.*, 2015 ▸). Various thia­zole-bearing compounds have shown activities such as anti-bacterial, anti-fungal, anti-inflammatory, anti-hypertensive, anti-HIV, anti-tumor, anti-filarial, anti-convulsant, herbicidal, insecticidal, schistosomicidal and anthelmintic (Bharti *et al.*, 2010 ▸). The synthesis of thia­zole derivatives by various methods and their biological evaluation have been described by several researchers and the thia­zole nucleus has therefore attracted a lot of inter­est for the development of pharmacologically active compounds (Breslow, 1958 ▸). In our studies, a new Schiff base, 4-{[(*E*)-4-(hept­yloxy)benzyl­idene]amino}-*N*-(naphthalen-2-yl)-1,3-thia­zol-2-amine, was obtained in crystalline form from the reaction of 2-amino-4-(2-naphth­yl)thia­zole with 4-*N*-(hept­yloxy)benzaldehyde. We report here the synthesis and the crystal and mol­ecular structures of the title compound, including a Hirshfeld surface analysis to assess the relative importance of the various inter­molecular inter­actions on the crystal packing.

## Structural commentary   

**Figure 1:**
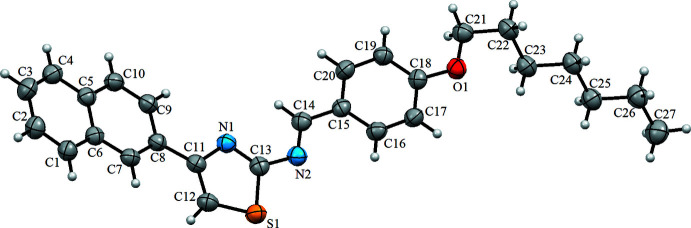
The mol­ecular structure of the title compound, with atom labelling. Displacement ellipsoids are drawn at the 40% probability level.

The asymmetric unit of the title compound contains one mol­ecule (Fig. 1 ▸). The naphthalene unit makes a dihedral angle of 14.24 (16)° with the thia­zole ring. The anisole ring is inclined to the thia­zole ring by a dihedral angle of 13.18 (22)°. The heptyl chain attached to O1 is twisted out of this plane with the O1—C21—C22–C23 torsion angle being 61.1 (4)°. In the thia­zole ring, the C11—N1 [1.373 (4) Å] and C13—N1 [1.298 (4) Å] distances indicate substantial electronic delocalization (Table 1 ▸).

## Supra­molecular features   

**Figure 2:**
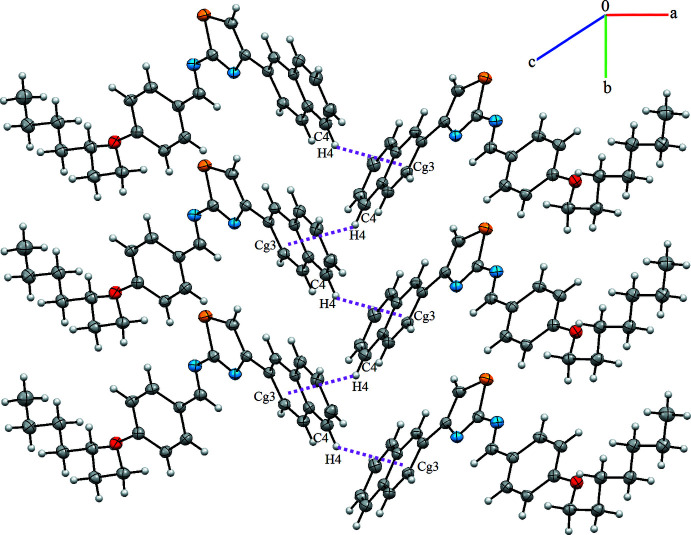
A view of the crystal packing of the title compound. The C—H⋯π(ring) inter­actions are indicated by dashed lines.

In the crystal, the most important inter­molecular contacts are C—H⋯π inter­actions, which link screw-related mol­ecules *via* C4—H4⋯*Cg*3^i^ [symmetry code: (i) −*x*, *y* + 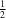, −*z* + 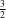), forming zigzag chains that extend parallel to the *b* axis (Fig. 2 ▸ and Table 2 ▸). The distance of between the carbon atom C4 and the centroid (*Cg*3^i^) of the adjacent C5-C10 ring is 3.522 (4) Å.

## Database survey   

A search of the Cambridge Structural Database (CSD, version 5.39; Groom *et al.*, 2016 ▸) for the (*E*)-1-(4-(hept­yloxy)phen­yl)-*N*-(4-(naphthalen-2-yl)thia­zol-2-yl)methanimine fragment revealed three hits. These structures are 4-(pyren-1-yl)-1,3-thia­zol-2-amine (pyrene thia­zole conjugate, PTC), C_19_H_12_N_2_S (SOPREW; Mahapatra *et al.*, 2014 ▸), 2-amino-4-(2-naphth­yl)-1,3-thia­zolium bromide, C_13_H_11_N_2_S^+^·Br^−^ (XUNKOG; Lynch *et al.*, 2002 ▸) and (*E*)-4-(4-chloro­phen­yl)-*N*-(1,3-benzodioxol-5-yl­methyl­ene)-5-(1*H*-1,2,4-triazol-1-yl)-1,3-thia­zol-2-amine, C_19_H_12_ClN_5_O_2_S (XAZJUE; Shao *et al.*, 2006 ▸). In XUNKOG, the mol­ecules are connected to each other *via* N—H⋯Br hydrogen bonds while in XAZJUE, they are linked by a weak C—H⋯O hydrogen bond. In SOPREW, the two pyrene thia­zole conjugate mol­ecules are connected into symmetrical homodimers by pairs of N—H⋯N hydrogen bonds.

## Hirshfeld surface analysis   

**Figure 3:**
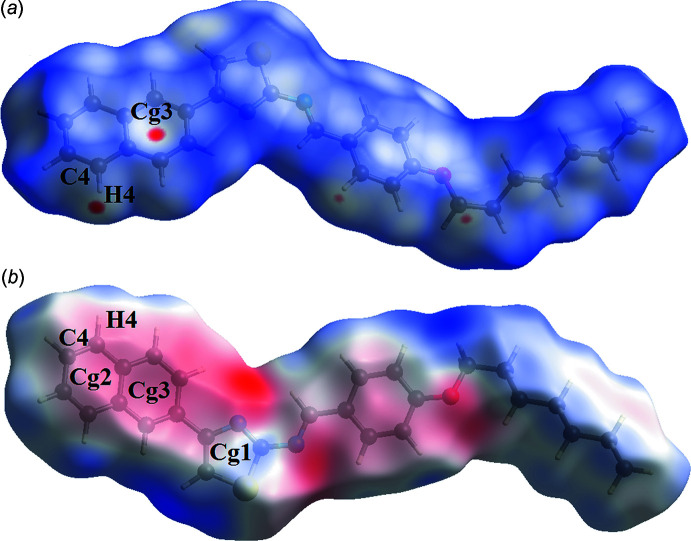
The Hirshfeld surfaces of the title compound mapped over (*a*) *d*_norm_, and (*b*) electrostatic potential.

**Figure 4:**
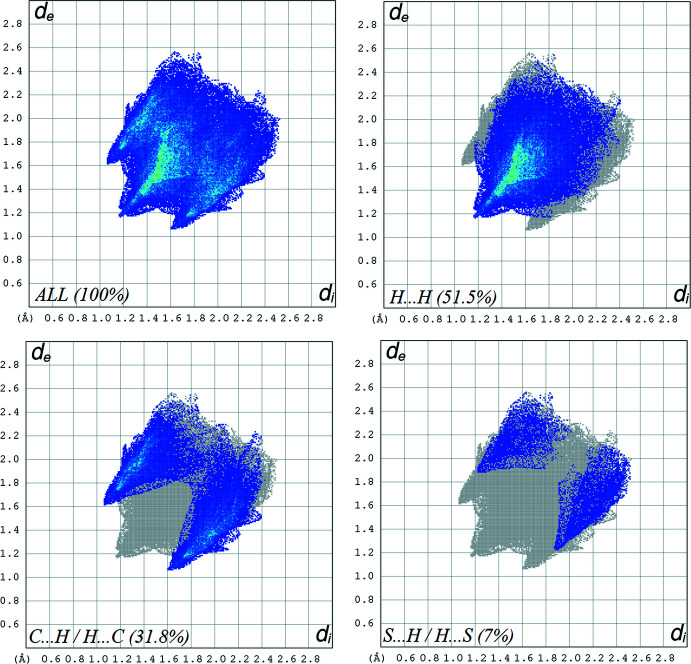
Two-dimensional fingerprint plots, showing the relative contribution of the atom-pair inter­actions to the Hirshfeld surface.

To investigate the inter­molecular inter­actions, Hirshfeld surface analysis (Spackman & Jayatilaka, 2009 ▸) and fingerprint plots were generated using *CrystalExplorer17.5* (Turner *et al.*, 2017 ▸). Hirshfeld surface analysis depicts inter­molecular inter­actions by different colours, representing short or long contacts, which reflect the relative strength of the inter­action. The generated Hirshfeld surface mapped over *d*_norm_ is shown in Fig. 3 ▸ *a* where the red spots correspond to the C—H⋯π(ring) close contacts (Table 2 ▸). The three-dimensional Hirshfeld surface plotted over electrostatic potential shows donor (red) and acceptor (blue) regions (Fig. 3 ▸ *b*). The crystal packing is dominated by H⋯H contacts, representing van der Waals inter­actions (51.5% contribution to the surface), followed by C⋯H/H⋯C and S⋯H/H⋯S inter­actions, which contribute 31.8% and 7%, respectively (Fig. 4 ▸).

## Synthesis and crystallization   

The title compound was prepared by adding 4-*N*-(hept­yloxy)benzaldehyde (0.1947 g, 0.885 mmol) dropwise to a constantly stirring solution of 2-amino-4-(2-naphth­yl)thia­zole (0.2 g, 0.885 mmol) in 1-propanol (10 ml). The reaction was catalysed by NaOH (0.1 g) and was stirred for 3 h in a water bath at 278–283 K. The reaction was monitored with thin-layer chromatography (TLC) using a 3:7 ratio of ethyl acetate to *n*-hexane (*R*_f_ = 0.775). The precipitate was filtered, washed with 1-propanol, and dried. The resulting solid was further purified by recrystallization from ethanol and diethyl ether. Single crystals of the title compound suitable for X-ray analysis were obtained by slow evaporation of an acetone solution (yield 81.7%, m.p. 387.5–389.5 K).

## Refinement   

Crystal data, data collection and structure refinement details are summarized in Table 3 ▸. The C-bound H atoms were placed in idealized positions and refined using a riding model: C—H = 0.93–0.97 Å with *U*_iso_(H) = 1.5*U*_eq_(C-meth­yl) and 1.2*U*_eq_(C) for other C-bound H atoms.

## Supplementary Material

Crystal structure: contains datablock(s) I. DOI: 10.1107/S2056989020007951/pk2631sup1.cif

Structure factors: contains datablock(s) I. DOI: 10.1107/S2056989020007951/pk2631Isup2.hkl

Supporting information file. DOI: 10.1107/S2056989020007951/pk2631Isup3.cml

CCDC reference: 1979809

Additional supporting information:  crystallographic information; 3D view; checkCIF report

## Figures and Tables

**Table 1:** Selected bond lengths (Å)

S1—C12	1.674 (4)	N1—C13	1.298 (4)
S1—C13	1.719 (4)	N1—C11	1.373 (4)
O1—C18	1.345 (4)	N2—C14	1.275 (4)
O1—C21	1.434 (4)	N2—C13	1.377 (4)

**Table 2:** Hydrogen-bond geometry (Å, °) *Cg*3 is the centroid of the C5–C10 ring.

*D*—H⋯*A*	*D*—H	H⋯*A*	*D*⋯*A*	*D*—H⋯*A*
C4—H4⋯*Cg*3^i^	0.93	2.85	3.522 (4)	130

Symmetry code: (i) 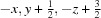.

**Table 3:** Experimental details

Crystal data	
Chemical formula	C_27_H_28_N_2_OS
*M*_r_	428.57
Crystal system, space group	Monoclinic, *P*2_1_/*c*
Temperature (K)	296
*a*, *b*, *c* (Å)	22.927 (4), 5.9315 (6), 17.191 (2)
β (°)	97.734 (12)
*V* (Å^3^)	2316.6 (5)
*Z*	4
Radiation type	Mo *K*α
μ (mm^−1^)	0.16
Crystal size (mm)	0.49 × 0.24 × 0.11

Data collection	
Diffractometer	Stoe IPDS 2
Absorption correction	Integration (*X-RED32*; Stoe & Cie, 2002 ▸)
*T*_min_, *T*_max_	0.955, 0.982
No. of measured, independent and observed [*I* > 2σ(*I*)] reflections	10986, 4068, 2000
*R*_int_	0.063
(sin θ/λ)_max_ (Å^−1^)	0.596

Refinement	
*R*[*F* ^2^ > 2σ(*F* ^2^)], *wR*(*F* ^2^), *S*	0.067, 0.155, 0.92
No. of reflections	4068
No. of parameters	281
H-atom treatment	H-atom parameters constrained
Δρ_max_, Δρ_min_ (e Å^−3^)	0.23, −0.17

Computer programs: *X-AREA* and *X-RED32* (Stoe & Cie, 2002 ▸), *SHELXT2017/1* (Sheldrick, 2015*a* ▸), *SHELXL2017/1* (Sheldrick, 2015*b* ▸), *PLATON* (Spek, 2020 ▸) and *WinGX* (Farrugia, 2012 ▸).

## supplementary crystallographic information

### Crystal data

**Table:**

C_27_H_28_N_2_OS	*F*(000) = 912
*M**_r_* = 428.57	*D*_x_ = 1.229 Mg m^−^^3^
Monoclinic, *P*2_1_/*c*	Mo *K*α radiation, λ = 0.71073 Å
*a* = 22.927 (4) Å	Cell parameters from 9222 reflections
*b* = 5.9315 (6) Å	θ = 1.4–27.7°
*c* = 17.191 (2) Å	µ = 0.16 mm^−^^1^
β = 97.734 (12)°	*T* = 296 K
*V* = 2316.6 (5) Å^3^	Stick, yellow
*Z* = 4	0.49 × 0.24 × 0.11 mm

### Data collection

**Table:**

Stoe IPDS 2 diffractometer	4068 independent reflections
Radiation source: sealed X-ray tube, 12 x 0.4 mm long-fine focus	2000 reflections with *I* > 2σ(*I*)
Plane graphite monochromator	*R*_int_ = 0.063
Detector resolution: 6.67 pixels mm^-1^	θ_max_ = 25.1°, θ_min_ = 2.4°
rotation method scans	*h* = −24→27
Absorption correction: integration (X-RED32; Stoe & Cie, 2002)	*k* = −7→7
*T*_min_ = 0.955, *T*_max_ = 0.982	*l* = −20→20
10986 measured reflections	

### Refinement

**Table:**

Refinement on *F*^2^	0 restraints
Least-squares matrix: full	Hydrogen site location: inferred from neighbouring sites
*R*[*F*^2^ > 2σ(*F*^2^)] = 0.067	H-atom parameters constrained
*wR*(*F*^2^) = 0.155	*w* = 1/[σ^2^(*F*_o_^2^) + (0.0697*P*)^2^] where *P* = (*F*_o_^2^ + 2*F*_c_^2^)/3
*S* = 0.92	(Δ/σ)_max_ < 0.001
4068 reflections	Δρ_max_ = 0.23 e Å^−^^3^
281 parameters	Δρ_min_ = −0.17 e Å^−^^3^

### Special details

**Table:**

Geometry. All esds (except the esd in the dihedral angle between two l.s. planes) are estimated using the full covariance matrix. The cell esds are taken into account individually in the estimation of esds in distances, angles and torsion angles; correlations between esds in cell parameters are only used when they are defined by crystal symmetry. An approximate (isotropic) treatment of cell esds is used for estimating esds involving l.s. planes.

### Fractional atomic coordinates and isotropic or equivalent isotropic displacement parameters (Å^2^)

**Table:**

	*x*	*y*	*z*	*U*_iso_*/*U*_eq_	
S1	0.22633 (5)	0.01514 (16)	0.57256 (6)	0.1138 (4)	
O1	0.34973 (11)	0.7781 (4)	0.18878 (14)	0.1090 (7)	
N1	0.17508 (13)	0.3977 (5)	0.56495 (16)	0.0951 (8)	
N2	0.23575 (14)	0.3219 (5)	0.46375 (17)	0.1016 (8)	
C6	0.09218 (14)	0.4575 (5)	0.81098 (19)	0.0907 (9)	
C7	0.12144 (15)	0.3353 (5)	0.7578 (2)	0.0922 (9)	
H7	0.138236	0.197033	0.773380	0.111*	
C18	0.31570 (16)	0.7233 (6)	0.24384 (19)	0.0942 (9)	
C8	0.12605 (15)	0.4127 (5)	0.68404 (19)	0.0893 (9)	
C13	0.20898 (16)	0.2713 (6)	0.52869 (19)	0.0957 (9)	
C15	0.25439 (16)	0.5758 (6)	0.36278 (18)	0.0911 (9)	
C9	0.09865 (16)	0.6188 (6)	0.6604 (2)	0.0983 (10)	
H9	0.100324	0.671600	0.609769	0.118*	
C16	0.29941 (17)	0.4482 (6)	0.33942 (19)	0.1012 (10)	
H16	0.309049	0.310612	0.363781	0.121*	
C5	0.06665 (15)	0.6676 (6)	0.7867 (2)	0.0957 (9)	
C19	0.27062 (16)	0.8513 (6)	0.2650 (2)	0.0987 (10)	
H19	0.260471	0.986613	0.239358	0.118*	
C11	0.16110 (15)	0.2922 (5)	0.63110 (19)	0.0905 (9)	
C17	0.32949 (17)	0.5193 (6)	0.28215 (19)	0.1011 (10)	
H17	0.359909	0.431246	0.267905	0.121*	
C20	0.24043 (16)	0.7789 (6)	0.3243 (2)	0.1000 (10)	
H20	0.210185	0.867189	0.338883	0.120*	
C10	0.07004 (16)	0.7411 (6)	0.7095 (2)	0.1021 (10)	
H10	0.052252	0.876263	0.692155	0.123*	
C1	0.08963 (16)	0.3841 (7)	0.8886 (2)	0.1035 (10)	
H1	0.105644	0.245213	0.905032	0.124*	
C14	0.22370 (16)	0.5061 (6)	0.42680 (19)	0.0977 (9)	
H14	0.194260	0.598284	0.441516	0.117*	
C12	0.18554 (17)	0.0833 (6)	0.6429 (2)	0.1043 (10)	
H12	0.180195	−0.009921	0.684812	0.125*	
C4	0.04009 (17)	0.7950 (7)	0.8408 (2)	0.1113 (11)	
H4	0.023035	0.933143	0.825662	0.134*	
C23	0.37841 (18)	0.7782 (7)	0.0293 (2)	0.1100 (11)	
H23A	0.385642	0.645391	0.062131	0.132*	
H23B	0.340038	0.760901	−0.001570	0.132*	
C21	0.33361 (18)	0.9678 (6)	0.1388 (2)	0.1106 (11)	
H21A	0.334233	1.104756	0.169733	0.133*	
H21B	0.294231	0.947554	0.111073	0.133*	
C25	0.42596 (19)	0.5902 (7)	−0.0784 (2)	0.1155 (12)	
H25A	0.432021	0.455243	−0.046493	0.139*	
H25B	0.388034	0.576225	−0.110681	0.139*	
C24	0.42408 (18)	0.7903 (7)	−0.0250 (2)	0.1128 (11)	
H24A	0.462375	0.806563	0.006187	0.135*	
H24B	0.417052	0.924663	−0.057046	0.135*	
C2	0.06433 (18)	0.5126 (8)	0.9391 (2)	0.1156 (11)	
H2	0.063744	0.463555	0.990321	0.139*	
C22	0.37726 (19)	0.9836 (7)	0.0817 (2)	0.1149 (11)	
H22A	0.416186	1.005154	0.110597	0.138*	
H22B	0.368155	1.115365	0.048834	0.138*	
C26	0.4728 (2)	0.6016 (8)	−0.1311 (2)	0.1277 (14)	
H26A	0.510749	0.615254	−0.098731	0.153*	
H26B	0.466844	0.736962	−0.162780	0.153*	
C3	0.0389 (2)	0.7194 (8)	0.9150 (3)	0.1247 (13)	
H3	0.021019	0.806070	0.950183	0.150*	
C27	0.4750 (2)	0.4030 (8)	−0.1848 (3)	0.1497 (17)	
H27A	0.438855	0.394620	−0.220161	0.225*	
H27B	0.480019	0.267150	−0.154273	0.225*	
H27C	0.507361	0.420161	−0.214350	0.225*	

### Atomic displacement parameters (Å^2^)

**Table:**

	*U*^11^	*U*^22^	*U*^33^	*U*^12^	*U*^13^	*U*^23^
S1	0.1338 (8)	0.0960 (6)	0.1147 (7)	0.0124 (6)	0.0282 (6)	0.0065 (5)
O1	0.1105 (17)	0.1179 (17)	0.1008 (15)	0.0099 (14)	0.0219 (14)	0.0124 (14)
N1	0.101 (2)	0.0915 (16)	0.0922 (17)	0.0001 (15)	0.0123 (15)	0.0032 (15)
N2	0.117 (2)	0.0974 (19)	0.0913 (17)	−0.0022 (17)	0.0169 (16)	0.0002 (16)
C6	0.085 (2)	0.091 (2)	0.094 (2)	−0.0032 (17)	0.0068 (17)	−0.0021 (18)
C7	0.095 (2)	0.0804 (19)	0.099 (2)	−0.0006 (17)	0.0065 (19)	0.0057 (17)
C18	0.096 (2)	0.102 (2)	0.0839 (19)	0.000 (2)	0.0095 (18)	−0.0011 (19)
C8	0.088 (2)	0.0826 (19)	0.097 (2)	−0.0055 (16)	0.0109 (18)	0.0054 (17)
C13	0.096 (2)	0.101 (2)	0.089 (2)	−0.0075 (19)	0.0092 (19)	−0.0060 (19)
C15	0.096 (2)	0.091 (2)	0.0856 (19)	0.0002 (18)	0.0093 (17)	−0.0069 (17)
C9	0.098 (2)	0.093 (2)	0.103 (2)	−0.0053 (19)	0.012 (2)	0.0107 (19)
C16	0.121 (3)	0.090 (2)	0.092 (2)	0.009 (2)	0.013 (2)	0.0028 (18)
C5	0.087 (2)	0.091 (2)	0.109 (3)	−0.0041 (18)	0.0102 (19)	−0.0008 (19)
C19	0.104 (3)	0.094 (2)	0.098 (2)	0.007 (2)	0.014 (2)	0.0085 (18)
C11	0.093 (2)	0.085 (2)	0.092 (2)	−0.0088 (18)	0.0082 (18)	0.0037 (17)
C17	0.113 (3)	0.097 (2)	0.095 (2)	0.016 (2)	0.018 (2)	−0.001 (2)
C20	0.101 (2)	0.096 (2)	0.102 (2)	0.0135 (19)	0.014 (2)	−0.003 (2)
C10	0.100 (2)	0.085 (2)	0.120 (3)	0.0019 (18)	0.015 (2)	0.010 (2)
C1	0.103 (3)	0.110 (2)	0.098 (2)	0.005 (2)	0.014 (2)	0.005 (2)
C14	0.103 (2)	0.096 (2)	0.093 (2)	−0.002 (2)	0.0103 (19)	−0.005 (2)
C12	0.116 (3)	0.091 (2)	0.106 (2)	0.001 (2)	0.012 (2)	0.0129 (18)
C4	0.110 (3)	0.103 (2)	0.121 (3)	0.006 (2)	0.015 (2)	−0.005 (2)
C23	0.109 (3)	0.117 (3)	0.104 (2)	−0.009 (2)	0.014 (2)	0.011 (2)
C21	0.122 (3)	0.102 (2)	0.107 (2)	−0.001 (2)	0.015 (2)	0.008 (2)
C25	0.120 (3)	0.126 (3)	0.100 (2)	−0.006 (2)	0.015 (2)	0.009 (2)
C24	0.113 (3)	0.126 (3)	0.100 (2)	−0.011 (2)	0.017 (2)	0.015 (2)
C2	0.112 (3)	0.133 (3)	0.101 (2)	0.008 (3)	0.013 (2)	0.001 (3)
C22	0.121 (3)	0.114 (3)	0.112 (2)	−0.010 (2)	0.023 (2)	0.015 (2)
C26	0.137 (4)	0.142 (3)	0.107 (3)	−0.009 (3)	0.026 (3)	0.003 (3)
C3	0.128 (3)	0.128 (3)	0.122 (3)	0.013 (3)	0.030 (3)	−0.017 (3)
C27	0.173 (5)	0.157 (4)	0.121 (3)	0.002 (3)	0.029 (3)	−0.014 (3)

### Geometric parameters (Å, º)

**Table:**

S1—C12	1.674 (4)	C10—H10	0.9300
S1—C13	1.719 (4)	C1—C2	1.343 (5)
O1—C18	1.345 (4)	C1—H1	0.9300
O1—C21	1.434 (4)	C14—H14	0.9300
N1—C13	1.298 (4)	C12—H12	0.9300
N1—C11	1.373 (4)	C4—C3	1.356 (5)
N2—C14	1.275 (4)	C4—H4	0.9300
N2—C13	1.377 (4)	C23—C24	1.495 (5)
C6—C7	1.406 (4)	C23—C22	1.517 (5)
C6—C1	1.413 (4)	C23—H23A	0.9700
C6—C5	1.416 (5)	C23—H23B	0.9700
C7—C8	1.366 (4)	C21—C22	1.497 (5)
C7—H7	0.9300	C21—H21A	0.9700
C18—C19	1.370 (5)	C21—H21B	0.9700
C18—C17	1.394 (5)	C25—C26	1.497 (5)
C8—C9	1.409 (5)	C25—C24	1.505 (5)
C8—C11	1.477 (4)	C25—H25A	0.9700
C15—C16	1.382 (5)	C25—H25B	0.9700
C15—C20	1.391 (5)	C24—H24A	0.9700
C15—C14	1.444 (5)	C24—H24B	0.9700
C9—C10	1.349 (4)	C2—C3	1.397 (6)
C9—H9	0.9300	C2—H2	0.9300
C16—C17	1.344 (5)	C22—H22A	0.9700
C16—H16	0.9300	C22—H22B	0.9700
C5—C4	1.400 (5)	C26—C27	1.502 (6)
C5—C10	1.409 (5)	C26—H26A	0.9700
C19—C20	1.376 (4)	C26—H26B	0.9700
C19—H19	0.9300	C3—H3	0.9300
C11—C12	1.364 (5)	C27—H27A	0.9600
C17—H17	0.9300	C27—H27B	0.9600
C20—H20	0.9300	C27—H27C	0.9600
			
C12—S1—C13	89.11 (18)	C11—C12—H12	124.1
C18—O1—C21	119.0 (3)	S1—C12—H12	124.1
C13—N1—C11	110.8 (3)	C3—C4—C5	120.8 (4)
C14—N2—C13	120.0 (3)	C3—C4—H4	119.6
C7—C6—C1	122.7 (3)	C5—C4—H4	119.6
C7—C6—C5	118.5 (3)	C24—C23—C22	113.7 (3)
C1—C6—C5	118.8 (3)	C24—C23—H23A	108.8
C8—C7—C6	122.3 (3)	C22—C23—H23A	108.8
C8—C7—H7	118.9	C24—C23—H23B	108.8
C6—C7—H7	118.9	C22—C23—H23B	108.8
O1—C18—C19	125.6 (3)	H23A—C23—H23B	107.7
O1—C18—C17	115.2 (3)	O1—C21—C22	107.5 (3)
C19—C18—C17	119.1 (3)	O1—C21—H21A	110.2
C7—C8—C9	118.2 (3)	C22—C21—H21A	110.2
C7—C8—C11	121.7 (3)	O1—C21—H21B	110.2
C9—C8—C11	120.0 (3)	C22—C21—H21B	110.2
N1—C13—N2	128.6 (3)	H21A—C21—H21B	108.5
N1—C13—S1	114.7 (2)	C26—C25—C24	114.6 (3)
N2—C13—S1	116.5 (3)	C26—C25—H25A	108.6
C16—C15—C20	118.0 (3)	C24—C25—H25A	108.6
C16—C15—C14	121.6 (3)	C26—C25—H25B	108.6
C20—C15—C14	120.4 (3)	C24—C25—H25B	108.6
C10—C9—C8	121.4 (3)	H25A—C25—H25B	107.6
C10—C9—H9	119.3	C23—C24—C25	115.0 (3)
C8—C9—H9	119.3	C23—C24—H24A	108.5
C17—C16—C15	121.2 (3)	C25—C24—H24A	108.5
C17—C16—H16	119.4	C23—C24—H24B	108.5
C15—C16—H16	119.4	C25—C24—H24B	108.5
C4—C5—C10	122.9 (3)	H24A—C24—H24B	107.5
C4—C5—C6	118.6 (3)	C1—C2—C3	120.4 (4)
C10—C5—C6	118.5 (3)	C1—C2—H2	119.8
C18—C19—C20	119.7 (3)	C3—C2—H2	119.8
C18—C19—H19	120.1	C21—C22—C23	113.9 (3)
C20—C19—H19	120.1	C21—C22—H22A	108.8
C12—C11—N1	113.6 (3)	C23—C22—H22A	108.8
C12—C11—C8	126.5 (3)	C21—C22—H22B	108.8
N1—C11—C8	119.8 (3)	C23—C22—H22B	108.8
C16—C17—C18	120.8 (3)	H22A—C22—H22B	107.7
C16—C17—H17	119.6	C25—C26—C27	115.0 (4)
C18—C17—H17	119.6	C25—C26—H26A	108.5
C19—C20—C15	121.1 (3)	C27—C26—H26A	108.5
C19—C20—H20	119.5	C25—C26—H26B	108.5
C15—C20—H20	119.5	C27—C26—H26B	108.5
C9—C10—C5	121.1 (3)	H26A—C26—H26B	107.5
C9—C10—H10	119.5	C4—C3—C2	120.6 (4)
C5—C10—H10	119.5	C4—C3—H3	119.7
C2—C1—C6	120.7 (4)	C2—C3—H3	119.7
C2—C1—H1	119.6	C26—C27—H27A	109.5
C6—C1—H1	119.6	C26—C27—H27B	109.5
N2—C14—C15	122.0 (3)	H27A—C27—H27B	109.5
N2—C14—H14	119.0	C26—C27—H27C	109.5
C15—C14—H14	119.0	H27A—C27—H27C	109.5
C11—C12—S1	111.8 (3)	H27B—C27—H27C	109.5
			
C1—C6—C7—C8	−176.9 (3)	C15—C16—C17—C18	−0.8 (5)
C5—C6—C7—C8	0.1 (5)	O1—C18—C17—C16	178.7 (3)
C21—O1—C18—C19	−9.9 (5)	C19—C18—C17—C16	−0.3 (5)
C21—O1—C18—C17	171.2 (3)	C18—C19—C20—C15	−0.8 (5)
C6—C7—C8—C9	−2.1 (5)	C16—C15—C20—C19	−0.3 (5)
C6—C7—C8—C11	174.8 (3)	C14—C15—C20—C19	177.8 (3)
C11—N1—C13—N2	−175.0 (3)	C8—C9—C10—C5	0.2 (5)
C11—N1—C13—S1	−0.1 (4)	C4—C5—C10—C9	176.6 (4)
C14—N2—C13—N1	−7.2 (6)	C6—C5—C10—C9	−2.3 (5)
C14—N2—C13—S1	178.0 (3)	C7—C6—C1—C2	175.9 (4)
C12—S1—C13—N1	0.2 (3)	C5—C6—C1—C2	−1.1 (5)
C12—S1—C13—N2	175.8 (3)	C13—N2—C14—C15	174.4 (3)
C7—C8—C9—C10	2.0 (5)	C16—C15—C14—N2	−1.4 (5)
C11—C8—C9—C10	−175.0 (3)	C20—C15—C14—N2	−179.4 (3)
C20—C15—C16—C17	1.0 (5)	N1—C11—C12—S1	0.2 (4)
C14—C15—C16—C17	−177.0 (3)	C8—C11—C12—S1	−175.5 (3)
C7—C6—C5—C4	−176.8 (3)	C13—S1—C12—C11	−0.2 (3)
C1—C6—C5—C4	0.3 (5)	C10—C5—C4—C3	−178.7 (4)
C7—C6—C5—C10	2.1 (5)	C6—C5—C4—C3	0.2 (5)
C1—C6—C5—C10	179.2 (3)	C18—O1—C21—C22	−177.7 (3)
O1—C18—C19—C20	−177.8 (3)	C22—C23—C24—C25	−179.3 (3)
C17—C18—C19—C20	1.1 (5)	C26—C25—C24—C23	−178.5 (3)
C13—N1—C11—C12	−0.1 (4)	C6—C1—C2—C3	1.4 (6)
C13—N1—C11—C8	176.0 (3)	O1—C21—C22—C23	61.1 (4)
C7—C8—C11—C12	9.5 (5)	C24—C23—C22—C21	−177.2 (3)
C9—C8—C11—C12	−173.7 (4)	C24—C25—C26—C27	−179.8 (4)
C7—C8—C11—N1	−166.1 (3)	C5—C4—C3—C2	0.1 (6)
C9—C8—C11—N1	10.8 (5)	C1—C2—C3—C4	−0.9 (6)

### Hydrogen-bond geometry (Å, º)

*Cg*3 is the centroid of the C5–C10 ring.

**Table:**

*D*—H···*A*	*D*—H	H···*A*	*D*···*A*	*D*—H···*A*
C4—H4···*Cg*3^i^	0.93	2.85	3.522 (4)	130

Symmetry code: (i) −*x*, *y*+1/2, −*z*+3/2.
